# Editorial: The Dark Side of Microscopic Colitis

**DOI:** 10.3389/fmed.2021.809136

**Published:** 2021-12-02

**Authors:** Laura Francesca Pisani, Gian Eugenio Tontini, Luca Pastorelli

**Affiliations:** ^1^Gastroenterology and Endoscopy Unit, Istituto di Ricovero e Cura a Carattere Scientifico (IRCCS) Policlinico San Donato, Milan, Italy; ^2^Department of Medical-Surgical Physiopathology and Transplantation, Università degli Studi di Milano, Milan, Italy; ^3^Gastroenterology and Endoscopy Unit, Istituto di Ricovero e Cura a Carattere Scientifico (IRCCS) Ca' Granda Ospedale Maggiore Policlinico di Milano, Milan, Italy; ^4^Gastroenterology and Liver Unit, ASST Santi Paolo e Carlo, Milan, Italy; ^5^Department of Health Sciences, Università degli Studi di Milano, Milan, Italy

**Keywords:** microscopic colitis, collagenous colitis, lymphocytic colitis, inflammation, inflammatory pathway, epidemiology

Microscopic colitis (MC) encompasses both collagenous colitis (CC) and lymphocytic colitis (LC). Patients report chronic, watery, non-bloody diarrhea, abdominal pain, weight loss, and fatigue that may impair health-related quality of life (1). Over the last few years, the incidence and prevalence of this condition has risen, partly due to both greater awareness and increasing numbers of patients being diagnosed (2–5). Diagnosis of MC is by colonoscopy, and histological analysis of multiple biopsy specimens collected along the colon is used to assess activity (6, 7). However, heterogeneous immune profiles and disease characteristics among patients still make the pathogenesis of MC unclear (8, 9). Consequently, there is an emerging need for simple non-invasive tools that are also rapid, convenient, standardized and reproducible, for correct diagnosis and appropriate management during follow-up (10, 11).

In this Research Topic, experts in the field provide updated information on epidemiology, risk factors and therapy. They also propose key points for the identification of biomarkers and explore the immunological pathways involved in the pathogenesis of MC.

There are conflicting data on the epidemiology and risks factors for MC. Newer estimates of incidence in Europe and North America range from 0.6 to 16.4 cases per 100,000 person-years for CC, and from 0.6 to 16.0 cases per 100,000 person-years for LC (12). The incidence was higher in women than in men and increased with older age, demonstrating a peak between 60 and 80 years of age, in particular in postmenopausal women on hormone replacement therapy and also in women using an oral contraceptive as reported by Mihaly et al. In their article, Oruganti et al. provide an overview of risk factors for MC identified in a cohort of 216 patients, confirming the association of MC with medications such as non-steroidal anti-inflammatory drugs (NSAIDs), proton pump inhibitors (PPIs), statins and hormonal therapies, highlighting as a new risk factor the use of tricyclic antidepressants commonly prescribed in patients with functional gastrointestinal disorders. Multiple prospective cohort and cross-sectional studies have shown a strong association between cigarette smoking and MC (odds ratio 2.12) (13) with a significantly higher risk among current smokers compared with never-smokers (OR of 2.99) (14). The risk attenuated among former smokers but remained significantly higher than never-smokers (14). No dietary factors have yet been shown to conclusively have an association between dietary factors and the risk of MC. Intakes of protein, carbohydrates, gluten, sucrose, saturated fat, monounsaturated fat, polyunsaturated fat, omega-3 or omega-6 fatty acids, fiber, and zinc were not associated with MC and also a diet quality index score based on adherence to dietary recommendations, failed to reveal any associated with risk of both CC and LC (15, 16). Only alcohol consumption was associated with increased risk of developing MC and risk appeared to be dose responsive and independent of other known risk factors for MC (13, 17, 18).

Treatment is guided by response to symptoms, with budesonide identified as the most effective drug to control MC (19–21). Unfortunately, as reported by Mihaly et al. not enough data have been received from extensive trials on alternative drugs including immunomodulators, aminosalicylates, and biological treatment such as TNF-alpha antagonists and integrin inhibitors.

Patients with MC have a decreased risk of developing inflammation-associated colorectal cancer compared with patients with ulcerative colitis and with healthy individuals (12, 22). The reason behind this difference in risk remains unknown. The immune system can detect and remove malignant cells and many immunomodulatory molecules, including those involved in immune checkpoints (23). Lushnikova et al. examined whether increased immune surveillance in patients with MC could partly explain the lower observed risk of developing colorectal cancer, and reported systemically and locally altered levels of immunomodulatory molecules in patients with MC compared with controls. Further studies are needed to provide insights into the underlying mechanisms behind the seemingly protective effects of MC against colorectal cancer.

A more obscure field in MC is pathogenesis, whereby a dysregulated immune response to changes in the gut luminal environment in predisposed individuals results in uncontrolled chronic inflammation (9). However, increasing evidence has allowed researchers to examine a few key pathogenic mechanisms in the fields of genetics, the adaptive and innate immune response, and microRNA (8). Several alterations in the balance of different lymphocyte populations and cytokine profiles have been described in MC. Immunohistochemical analysis showed that lamina propria CD4^+^ T cells expressed the Th2 transcription factor GATA-3, whereas lamina propria CD8^+^ T cells expressed both GATA-3 and the Th1 transcription factor T-bet (24). Flow cytometry analyses and immunohistochemistry studies have shown heavy infiltration of CD8^+^ T cells in the mucosa of patients with CC and LC (25, 26). In their case report, Honjo et al. employed immunohistochemistry to analyse the association between disease activity and type of immune cell in the colonic mucosa, and found the active disease phase was characterized by the accumulation of CD3^+^ T cells, CD4^+^ T cells, CD8^+^ T cells, FOXP3^+^ Tregs, and CD68^+^ macrophages in the colonic lamina propria. Paradoxically, induction of remission was accompanied by a marked reduction in the accumulation of FOXP3^+^ Tregs in the colonic mucosa.

Despite the huge expansion in the knowledge of MC, we are still far from understanding the full spectrum of events leading to LC and CC. One basis of uncertainty is the fact that forms of MC can transform into each other and into classical inflammatory bowel disease, and that genetic similarities with IBD can be detected. One of the largest obstacles is the overwhelming complexity of diagnosis, treatment and management. In order to move forward, it is important to integrate the huge amount of basic and clinical data available in order to provide insights into the complexity and variability of MC. In few years, we expect that sharing of big data and machine-learning algorithms will integrate the wealth of available preclinical and clinical data with omics data (e.g., genomics, transcriptomics, epigenomics, proteomics, metabolomics), shedding new light on MC and promoting an improved and personalized clinical approach to patients. Overall, we are very pleased to have worked on this Research Topic. All the authors have provided innovative and outstanding contributions which will certainly stimulate new research and advances in the MC field.

## Author Contributions

LFP, GT, and LP conceived and wrote the editorial, approved the final version, and fully agree with its content.

## Conflict of Interest

The authors declare that the research was conducted in the absence of any commercial or financial relationships that could be construed as a potential conflict of interest.

## Publisher's Note

All claims expressed in this article are solely those of the authors and do not necessarily represent those of their affiliated organizations, or those of the publisher, the editors and the reviewers. Any product that may be evaluated in this article, or claim that may be made by its manufacturer, is not guaranteed or endorsed by the publisher.
